# Application of Structural Interaction Fingerpints (SIFts) into post-docking analysis - insight into activity and selectivity

**DOI:** 10.1186/1758-2946-5-S1-P28

**Published:** 2013-03-22

**Authors:** Jagna Witek, Krzysztof Rataj, Stefan Mordalski, Sabina Smusz, Tomasz Kosciolek, Andrzej J Bojarski

**Affiliations:** 1 Department of Medicinal Chemistry, Institute of Pharmacology Polish Academy of Sciences, 12 Smętna Street, 31-343 Kraków, Poland

## 

Cheminformatic methods, such as Virtual Screening, constitute a vital part of modern drug design process. This technique enables not only viable prediction of physicochemical properties of the molecules, but also effective database mining, being particularly useful tool in search for ligands of desired activity. Successful performance in case of single target drugs, implies a potential to extend its capabilities to compounds bearing desired activity towards multiple receptors.

In this research, we present application of Structural Interaction Fingerprints (SIFts) [1] combined with Machine Learning (ML) as a method to select single- and multi-target ligands from the docking results. A handful of protein kinases pairs was designated as targets. Collection of pseudo selective compounds, with various activity profiles, was acquired from ChEMBL database. Furthermore, for each target a set of ligands of various activity was aggregated. Decoy structures were random ligands from ZINC database. The compounds were docked into respective proteins, and SIFts were calculated for each protein-ligand complex. Training sets used in ML experiments consisted of cluster centroids of active and inactive ligands, whereas test sets were composed of remaining compounds, that returned docking poses.

The key aim of this study is to develop a viable method to filter the docking results, so that the compounds meeting desired activity profile are selected.
